# Silver Nanoparticles: A Promising Antifungal Agent against the Growth and Biofilm Formation of the Emergent *Candida auris*

**DOI:** 10.3390/jof8070744

**Published:** 2022-07-19

**Authors:** Reem AlJindan, Doaa M. AlEraky

**Affiliations:** 1 Department of Microbiology, College of Medicine, Imam Abdulrahman Bin Faisal University, Dammam P.O. Box 1982, Saudi Arabia; raljindan@iau.edu.sa; 2 Department of Biomedical Dental Science, Microbiology and Immunology, College of Dentistry, Imam Abdulrahman Bin Faisal University, Dammam P.O. Box 1982, Saudi Arabia

**Keywords:** *Candida auris*, emerging pathogen, antifungal susceptibility testing, anti-biofilm formation

## Abstract

*Candida auris* is a globally-emerging pathogen that is correlated to nosocomial infections and high mortality rates, causing major outbreaks in hospitals and serious public health concerns worldwide. This study investigated the antifungal activity of silver nanoparticles (AgNPs) on clinical isolates of *C. auris.* A total of eight clinical isolates were collected from blood, urine, ear swab, and groin. *C. auris* was confirmed by MALDI-TOF MS, and gene sequencing. All isolates confirmed as *C. auris* were subjected to antimicrobial agents, including amphotericin B, fluconazole, caspofungin, voriconazole, micafungin, and flucytosine. A serial dilution of a silver nanoparticles solution was prepared to test antifungal susceptibility testing under planktonic conditions. Moreover, an antibiofilm activity assay was determined using a colony-forming assay and a cell viability assay by a live–dead yeast kit. Significant antifungal and antibiofilm activity of AgNPs was detected against all isolates; MIC was <6.25 μg/mL, the range of MFC was from 6.25 to 12.5 μg/mL for all isolates, and the highest value of IC_50_ was 3.2 μg/mL. Silver nanomaterials could represent a possible antimicrobial agent to prevent outbreaks caused by *C. auris* infections.

## 1. Introduction

*Candida* species is one of the most frequent infections in human beings. These infections result in a global threat with a more than 60% mortality rate. *Candida auris* is an emerging fungus that has been noticed worldwide with a significant fatality rate and a challenging nosocomial infection [1,2]. *C. auris* was first recently described from a culture of the external ear canal, then it was frequently isolated from blood and the respiratory and urinary tracts to transmit rapidly and persevere on the surfaces of hospital settings [1,2,3]. In Saudi Arabia, the first two cases were observed in 2018, then new cases of *C. auris* infection from different hospitals were described [4,5,6]. This emerging pathogen is frequently isolated from patients with various medical device infections, such as catheters, cardiac-implanted devices, and percutaneous feeding tubes [7].

The dilemma of *C. auris* detection for clinical microbiology laboratories results from its close correlation to other *Candida* species [8,9]. The emerging yeast (*C. auris)* grows on Sabouraud Dextrose Agar (SDA) and CHROMagar *Candida* medium at 37 °C and 42 °C. Moreover, MALDI-TOF MS and advanced molecular techniques are used as efficient methods for confirmation, yet both are expensive techniques [1,3,10].

The biofilm formation of *C. auris* exhibits growth in synthetic media with burdens greater than *C. albicans*; therefore, *C. auris* plays a major role in its persistence in healthcare settings [10]. Biofilm formation is expected to be implicated in *C. auris* infections and is involved in different medical device infections. Recent reports of *C. auris* indicated high rates of catheters as the sources of bloodstream infections, consistent with biofilm’s role in pathogenesis [11].

The three main classes of clinically used antifungal agents are amphotericin B, azoles, and echinocandins; however, ERG11 gene mutations increase the resistance against fluconazole. In contrast, echinocandin resistance is low and the antifungal susceptibility testing of *C. auris* is assessed by using microdilution or disk diffusion test methods [12,13]. The unpredictable antifungal resistance profile negatively impacts the treatment’s effectiveness. Furthermore, yeast can form biofilms; biofilms withstand exposure to high temperatures, quaternary ammonium compounds, and UV light. It can survive on plastic surfaces for several weeks. There are few reports exploring novel antifungal agents on *C. auris* clinical isolates, which is crucial to controlling *C. auris* infections. Silver nanotechnology can provide a promising cost-effective antimicrobial agent with a broad spectrum effect, including various *Candida* species [10,14,15,16].

The aim of this study was to investigate the effect of silver nanoparticles on clinical isolates of *C. auris* as an effective antifungal and antibiofilm agent against this global emerging pathogen.

## 2. Materials and Methods

### 2.1. Ethics Approval

The study was conducted according to the ethical guidelines and approved by the Microbiology Department, College of Medicine, and Institutional Review Board at Imam Abdulrahman bin Faisal University (approval number: 2021-01-015).

### 2.2. Collection of Samples and Isolation

A total of eight clinical samples were isolated and reported previously by our group [6]. Briefly, the samples were collected from different sites of infections from patients who were admitted to the hospital and identified using MALDI-TOF MS and were adapted to grow at 42 °C. The DNA extraction was performed to sequence the 18S rRNA gene and internal transcribed spacer 2 (ITS2) regions, then analyzed using BLASTn and UNITE programs. A stock solution of each isolate was stored at −80 °C [17].

### 2.3. Antifungal Susceptibility Profile

The assay of the eight isolates was performed using VITEK^®^ 2 AST-YS08 (bioMérieux, Marcy-l’Étoile, France), according to the manufacturer’s guidelines. Briefly, the inoculum was adjusted to 0.5 McFarland, the AST-YS08 card comprised serial dilution of antifungal concentrations, susceptibility to amphotericin B, fluconazole, voriconazole, caspofungin, micafungin, and flucytosine was assessed (0.25–16 μg/mL, 0.5–64 μg/mL, 0.12–8 μg/mL, 0.06–8 μg/mL, 0.12–8 μg/mL, and 1–64 μg/mL, respectively. Interpretation of MIC breakpoints was assessed by CDC recommendations: amphotericin B ≥ 2 μg/mL; fluconazole  ≥ 32 μg/mL; caspofungin  ≥ 2 μg/mL; micafungin  ≥ 4 μg/mL [18].

### 2.4. Silver Nanoparticles Preparation

A solution of silver nanoparticles (AgNPs) was provided by Dr. Yasser AlBadry. The synthesis of AgNPs coated with polyvinylpyrrolidone (PVP) was performed using a chemical reduction protocol with microwave-assisted synthesis according to Pal el. Al. Briefly, A 25 mL conical flask was prepared with 10 mL of 1% (*w*/*v*) ethanolic solution of PVP and 0.2 mL of 0.1 M AgNO_3_ and placed in a microwave. The process was operated at 100% power of 800 W and a frequency of 2450 MHz for 5 s. Finally, the pale yellow color indicated the formation of silver nanoparticles [19]. The size of the spherical silver nanoparticles in our study was 15–20 nm.

### 2.5. Antifungal Susceptibility Testing

The test was established according to the CLSI M27 protocol guidelines for *Candida* species, with few modifications [20]. Briefly, the isolates were sub-cultured and then washed twice with saline to be adjusted to 0.5 MacFarland. Then, 50 μL of each isolate was inoculated in 96-well plates (Thermo Fisher Scientific, Waltham, MA, USA). A two-fold serial dilution of silver nanoparticle dilution was prepared from 3.125 to 200 μg/mL. Then 50 μL of each dilution series was added to wells and incubated for 48 h at 35 °C. To detect the minimal inhibitory concentration (MIC), it was established as the concentration with no turbidity (microbial growth). Whereas the minimal fungicidal concentration (MFC) was recognized by inoculating 10 μL from each well on SDA agar plates and then overnight incubation at 37 °C. MFC was the lowest concentration at which the growth of *C. auris* was less than or equal to 2 colony-forming units (CFUs) [21].

### 2.6. Antibiofilm Activity Evaluation

Overnight cultures of *C. auris* were adjusted to 2 × 10^6^ cells/mL. A volume of 50 μL of the adjusted cell suspension was inoculated in microplates (Thermo Fisher Scientific, Waltham, MA, USA). Then, 50 μL of the two-fold dilution series of AgNPs was added for a concentration range from 3.125 to 200 μg/mL and plates were incubated at 37 °C for 48 h to allow biofilm formation, with positive and negative controls. The anti-biofilm effect of AgNPs was determined using the colony forming unit assay and cell viability assay [15].

A volume of 100 μL of each well was inoculated on SDA media and incubated at 37 °C The counting of colonies was performed within 24 h to easily distinguish the colonies before they overgrew and after 48 to 72 h to allow scoring of any slow grow isolates. The experiment was independently executed using 2 replicates of microplates and 3 replicates of the treatments to confirm the reproducibility.

The LIVE/DEAD™ yeast viability kit was executed as per the manufacturer’s instructions (Thermo Fisher Scientific, Waltham, MA, USA). A novel two fluorescent probes were used for fungal viability; plasma membrane integrity and metabolic activity of fungi were fundamental to convert the yellow–green–fluorescent i of FUN^®^ 1 into red/orange intravacuolar structures. A fluorescence microscope equipped with the DP-72 digital camera was used for the microscopic observation to detect the viability of cells [22,23].

Dose–response curves were generated from the collected data to assess the required concentration to reduce the biofilm activity by 50% (IC50 values) using Prism 8 (GraphPad Software, San Diego, CA, USA).

## 3. Results

The antifungal susceptibility assay is summarized in Table 1. The MICs of the six antifungals were tested against eight *C. auris* isolates. Fluconazole demonstrated no activity against five isolates, whereas three isolates of *C. auris* were resistant to amphotericin B. On the contrary, caspofungin and micafungin could be efficient antifungal activity.

The antifungal susceptibility testing of silver nanoparticles under planktonic conditions of *C. auris* isolates showed significant antimicrobial activity against all *C. auris* isolates, the MIC of AgNPs was <6.25 μg/mL, and the MFC was 12.5 μg/mL for all isolates, except one isolate was 6.25 μg/mL.

**Figure 1:**
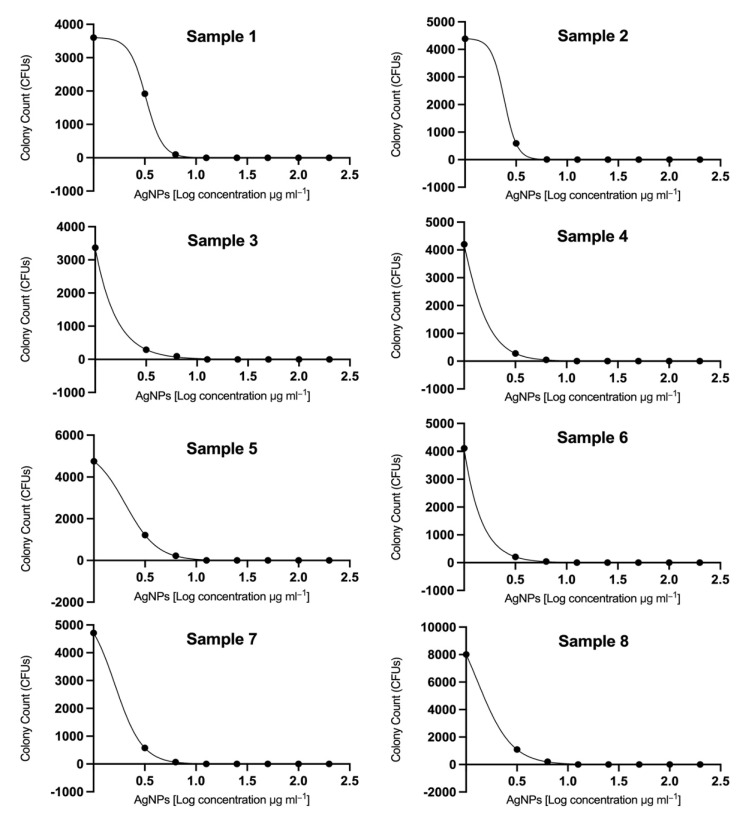
Silver nanoparticles (AgNPs) displayed effective inhibitory activity against eight *C. auris* clinical isolates. The concentrations of AgNPs ranged from 3.125 to 200 µg mL^−1^. The dose–response curves indicate that AgNPs displayed remarkable antibiofilm effects against *C. auris* isolates. The ranges of calculated IC_50_ values were determined from 0.7 to 3.2 µg/mL, where 5 out of 8 strains were less than 2 μg/mL.

**Figure 2:**
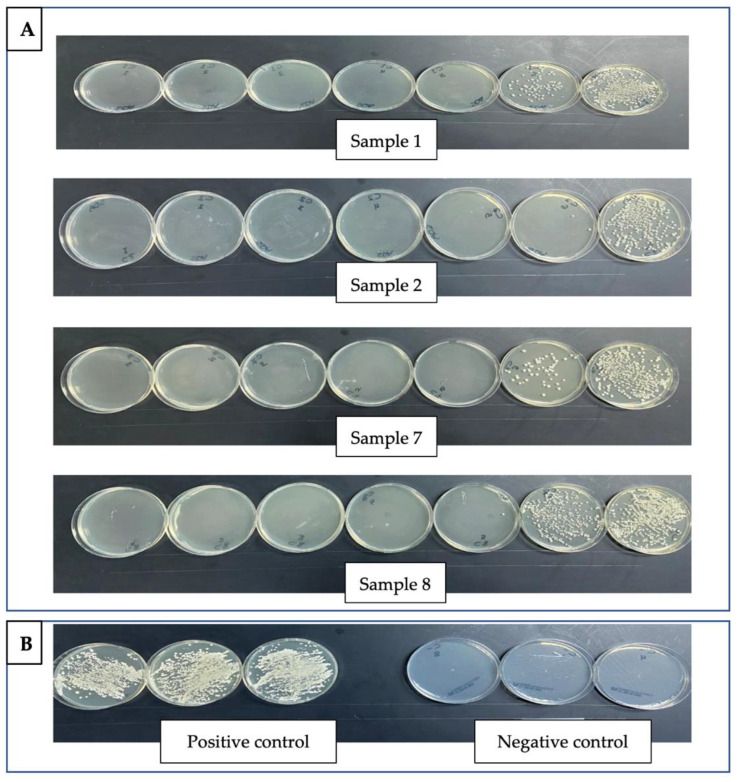
Silver nanoparticles (AgNPs) inhibit the biofilm formation on *C. auris* clinical isolates. (**A**). Four representative isolates (Sample nos. 1, 2, 7, and 8) and the serial dilution of silver nanoparticles from right to left corresponding from 3.125 to 200 μg/mL. (**B**). Positive and negative control samples. The colony-forming assay shows that AgNPs display effective inhibitory activity against the *C. auris* isolates.

**Figure 3:**
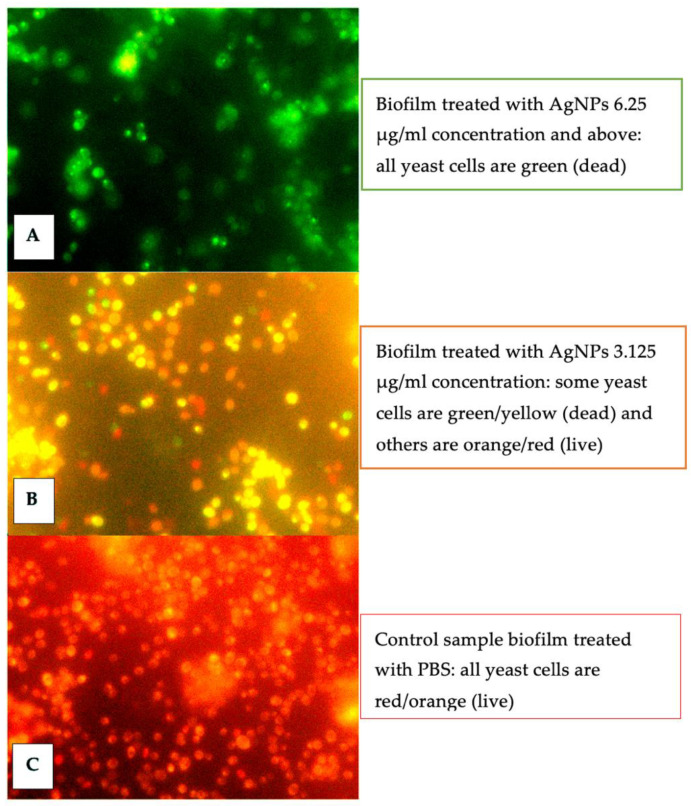
Fluorescence microscope images of the *C. auris* viability assay using the Live/Dead yeast kit (Thermo Fisher Scientific, Waltham, MA, USA); red/orange indicates live cells; green/yellow indicates dead cells. The figure shows a noticeable variation in the cell viability of silver nanoparticle-treated biofilm. (**A**) Biofilm treated with 6.25 μg/mL showed green/yellow yeast cells (**B**) Biofilm treated with 3.125 μg/mL showed cells in green/yellow and orange/red (**C**) Untreated biofilm samples showed orange/red cells.

The effect on biofilm formation was demonstrated in Figure 1, Figure 2 and Figure 3. The IC_50_ was calculated using Prism 8 (GraphPad Software, Inc.). A total of five out of eight strains showed IC_50_ less than 2 μg/mL (Figure 1). The attached cell forming the biofilm was assessed using colony forming units (CFUs) of *C. auris* isolates (Figure 2); silver nanoparticles presented a remarkable effect on the biofilm formation in all isolates.

The cell viability assay using the LIVE/DEAD™ yeast viability kit differentiated between the attached biofilm live and dead yeast cells with red and green fluorescence, respectively. The images of the fluorescence microscope showed red/orange cells and green/yellow, which indicated live cells and dead cells, respectively. The biofilm treated with 6.25 μg/mL only showed green/yellow yeast cells, the biofilm treated with 3.125 μg/mL showed different cells in green/yellow and orange/red, and finally, the untreated biofilms (PBS sample) showed orange/red cells only. These results also confirmed the potent effect of AgNPs (Figure 3). These results showed considerable activity of AgNPs to prevent biofilm formation in different clinical isolates of *C. auris.*

## 4. Discussion

The Centers for Disease Control and Prevention (CDC) has recognized *Candida auris* as an emerging fungal infection [24]. The prophylactic antifungal treatment is crucial to decrease the serious invasive infections that may progress after the colonization of yeast; therefore, CDC guidelines and recent literature suggest that treatment should be considered in patients with *C. auris* colonization [14,18]. Echinocandin is still an empirical treatment before antifungal sensitivity testing due to the frequent resistance to fluconazole. However global reports of increased resistance to echinocandins are a major concern [25]. One of the dilemmas of *C. auris* infection is the frequent resistance to antifungals even under planktonic conditions. Almost all isolates exhibit triazole resistance, and approximately 40% are multidrug-resistant [1,9,10,26].

Our results indicated that only three *C. auris* clinical isolates were resistant to amphotericin B, yet caspofungin and micafungin showed adequate antifungal activity. The resistance-associated mutations (Y132F and K143R) were reported previously, which justified the azole resistance [6]. The first cases of *C. auris* infection were described in Saudi Arabia, almost 4 years ago; later in 2020, another study reported an elevated rate of mortality in seven *C. auris* cases [4,27]. Other studies from Kuwait and Oman also reported high mortality rates and multidrug resistance among different cases of *C. auris* infections [28,29,30].

In our study, AgNPs showed remarkable antifungal effects against the growth of eight *C. auris* isolates, the MIC of AgNPs was <6.25 μg/mL, whereas MFC was 12.5 μg/mL for all isolates, except one isolate was 6.25 μg/mL. Our finding concurs with a study by Munoz. et al., who tested the effects of AgNPs against 10 strains and determined MIC values < 0.5 μg/mL; however, the range of MFC was from 1 to 2 µg/mL for 90% of strains, and only one strain showed higher MFC of 32 µg/mL [15].

The strains of *C. auris* can tolerate high temperatures and osmotic stress and have a great ability to produce many lytic enzymes and biofilm. Therefore, *C. auris* shows longer periods of survival compared to *C. albicans* on synthetic media, including plastics, metals, and dry conditions up to 14 days of survival. Therefore, antibiofilm activity is crucial to control biofilm formation on medical devices and surfaces and to prevent outbreaks in healthcare settings [3,26]. Although caspofungin is commonly effective against *Candida* biofilms, in a study reported by Sherry et al., caspofungin was ineffective against *C. auris* biofilms; on the contrary, chlorhexidine was recommended to inhibit planktonic communities of *C. auris* [10].

A recent study evaluated the effect of AgNPs on the CDC strain (*C. auris* 0390); the results of this study indicated that AgNPs concentrations from 2.3 to 0.017 ppm inhibited more than 80% of biofilm formation in dressings loaded with AgNPs [31]. In contrast, the effect of antibiofilm activity in our study was evaluated and more than 80% of biofilm formation was inhibited at a higher AgNP concentration of 6.25 μg/mL.

Additionally, AgNPs inhibited the biofilm formation effectively with a range of IC_50_ values from 0.7 to 3.2 µg/mL, where 5 out of 8 strains were <2 μg/mL, in contrast to a previous study that reported the calculated IC_50_ value was <2 μg/mL in 9 out of 10 strains [15].

To the best of our knowledge, this is the first study that evaluated the antibiofilm activity of clinical isolates using the cell viability assay, using the LIVE/DEAD™ yeast viability kit. The images by a fluorescence microscope confirmed the potent antibiofilm effect of AgNPs at a concentration of 6.25 μg/mL and the moderate effect at a concentration of 3.125 μg/mL compared to the biofilm treated with PBS (control group).

There were two salient features of this study. The first involved detecting the antimicrobial susceptibility testing of *C. auris* from patients with fungal infection and colonization. The second involved the antifungal susceptibility, determining the MIC and MFC of silver nanoparticles, and evaluating anti-biofilm activity using CFUs and a fluorescence microscope. The limitations of this study involved the insufficient quantity of silver nanoparticles to test other *Candida* species and the lack of access to the advanced scanning electron microscope (SEM) to visualize the antibiofilm effects of silver nanoparticles on *C. auris* isolates.

Although AgNPs showed remarkable effects against an emerging fungus, the debate on AgNP toxicity is still a major challenge [32,33]. A recent study reported that the effectiveness of the antimicrobial activities of AgNPs was correlated to the physicochemical properties, despite the method of synthesis. The study also concluded that, by applying a specific stabilizing agent, the selectivity of AgNP toxicity can be directed toward the desired pathogen [34].

There is a need for more studies to confer AgNPs at less toxicity, to be used in numerous biomedical applications and infection control. Further research studies are crucial to assess the use of AgNPs combined with antifungal therapy in humans, such as wound dressings, or as part of the disinfection strategies to reduce biofilm formation in hospitals and healthcare settings.

## 5. Conclusions

Silver nanomaterials (AgNPs) showed both inhibitory effects on the growth of *C. auris* and antibiofilm formation activity. The value of MIC was <6.25 μg/mL, whereas MFC was 12.5 μg/mL for all isolates, except one isolate was 6.25 μg/mL. More than 80% of biofilm formation was inhibited at a relatively high AgNP concentration (6.25 μg/mL), and the value of IC_50_ was determined from 0.7 to 3.2 µg/mL, where five out of eight strains were <2 μg/mL. Our findings were confirmed by a fluorescence microscope and indicate a potent antibiofilm effect of AgNPs at a concentration of 6.25 μg/mL compared to the biofilm treated with PBS (Control group). We conclude that silver nanoparticles could be used to control nosocomial infections and outbreaks in health institutes caused by *C. auris* infections.

## Figures and Tables

**Table 1:** Antifungal susceptibility testing of *C. auris* isolates.

Antifungal Susceptibility of *C. auris* MIC (ug/mL)								
Antifungal Agents	Sample 1	Sample 2	Sample 3	Sample 4	Sample 5	Sample 6	Sample 7	Sample 8
Amphotericin B	0.5	0.25	8	0.5	8	0.5	8	0.5
Fluconazole	16	16	32	32	32	32	32	8
Voriconazole	0.5	1	0.5	0.5	1	1	1	8
Caspofungin	0.25	0.25	0.25	0.25	0.25	0.25	0.25	0.25
Micafungin	0.06	0.06	0.06	0.06	0.06	0.06	0.06	0.06
Flucytosine	1	1	1	1	1	1	1	1

## Data Availability

Data and materials have been provided in the main manuscript, and where necessary, additional information about the study can be made available from the corresponding author upon reasonable request.

## Abbreviations

The following abbreviations are used in this manuscript:

*C. auris*	*Candida auris*
*C. albicans*	*Candida albicans*
MALDI TOF MS	matrix-assisted laser desorption/ionization-time of flight mass spectrometry
IPP	Infection Prevention Program
CDC	Centers for Disease Control and prevention
ITS	internal transcribed spacer
